# A Theranostic Nanoprobe for Hypoxia Imaging and Photodynamic Tumor Therapy

**DOI:** 10.3389/fchem.2019.00868

**Published:** 2019-12-20

**Authors:** Jing Hao Fan, Gui Ling Fan, Ping Yuan, Fu An Deng, Ling Shan Liu, Xiang Zhou, Xi Yong Yu, Hong Cheng, Shi Ying Li

**Affiliations:** ^1^Key Laboratory of Molecular Target & Clinical Pharmacology and The State Key Laboratory of Respiratory Disease, School of Pharmaceutical Sciences & The Fifth Affiliated Hospital, Guangzhou Medical University, Guangzhou, China; ^2^Guangdong Provincial Key Laboratory of Construction and Detection in Tissue Engineering, Biomaterials Research Center, School of Biomedical Engineering, Southern Medical University, Guangzhou, China

**Keywords:** hypoxia, photodynamic therapy, FRET, nanoprobe, theranostic

## Abstract

Hypoxia is a common feature for most malignant tumors, which was also closely related to the oxygen-dependent photodynamic therapy. Based on Förster resonance energy transfer (FRET), a smart nanoprobe (designated as H-Probe) was designed in this paper for hypoxia imaging and photodynamic tumor therapy. Due to the FRET process, H-Probe could respond to hypoxia with a significant fluorescence recovery. Moreover, abundant *in vitro* investigations demonstrated that the photosensitizer of PpIX in H-Probe could generate large amounts of singlet oxygen to kill cancer cells in the presence of oxygen and light with appropriate wavelength. Also, intravenously injected H-Probe with light irradiation achieved an effective tumor inhibition *in vivo* with a reduced side effect. This original strategy of integrating hypoxia imaging and tumor therapy in one nanoplatform would promote the development of theranostic nanoplatform for tumor precision therapy.

## Introduction

The malignant tumor progression induced abnormal microenvironment would induce therapeutic resistance and poor prognosis (Dalton, 1999; Whiteside, 2008; Kessenbrock et al., 2010; Sun et al., 2012; Gajewski et al., 2013; Liang et al., 2014; Sun, 2016). Among which, the fast proliferation of cancer cells and neovascularization deficiency would cause tumor hypoxia, which is a feature of most malignant tumors (Zhou et al., 2006; Conley et al., 2011; Rankin and Giaccia, 2016; Li S. Y. et al., 2018a). Moreover, the tumor heterogeneity and individual diversity of hypoxia microenvironments could further restrict the therapeutic outcome for personalized cancer treatment. Thus, the pre-evaluation for tumor hypoxia microenvironment is of great importance for cancer precision therapy (Li S. Y. et al., 2018b). To date, a variety of fluorescent probes has been developed for tumor hypoxia imaging (Zhang et al., 2010; Cui et al., 2011; Piao et al., 2013; Liu et al., 2014, 2017; Cai et al., 2015; Zheng et al., 2015; Yu et al., 2017). However, the separate implementation of imaging and therapeutic procedures may cause report delay due to the differences in distribution of the probe and drug. Therefore, it is meaningful to design a smart platform for simultaneous tumor hypoxia imaging and therapy.

Photodynamic therapy is known to be one of the non-invasive method for highly efficient tumor treatment (Dolmans et al., 2003; Castano et al., 2006; Chatterjee et al., 2008; Lucky et al., 2015; Li et al., 2017). Under the irradiation of light with appropriate wavelength, the photosensitizer in its ground state translated into excited state, which could excite the triplet oxygen into singlet oxygen with the energy transfer (Celli et al., 2010; Ethirajan et al., 2011; Li X. et al., 2018; Cheng et al., 2019a). Singlet oxygen as one of the main cytotoxic substances could trigger irreversible damage of various cell constituents (Li et al., 2016; Shen et al., 2016; Cheng et al., 2019b). Significantly, oxygen is indispensable during the photodynamic therapy, which would directly impact the therapeutic efficiency (Cheng et al., 2016). Considering that most tumors were hypoxic, one platform integrating hypoxia and photodynamic therapy would be instructive for tumor theranostics.

**Scheme 1:**
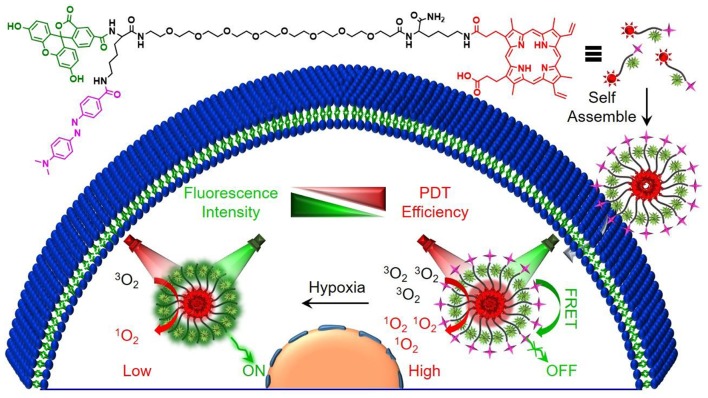
Schematic illustration of the preparation process and the proposed mechanism of H-Probe for hypoxia imaging and photodynamic tumor therapy.

In this work, a Förster resonance energy transfer (FRET)-based theranostic nanoprobe (designated as H-Probe) was designed for hypoxia imaging and photodynamic tumor therapy. As illustrated in Scheme 1, H-probe was made up of the hydrophilic PEG linker with the photosensitizer protoporphyrin IX (PpIX) and the FAM/Dabcyl-based FRET donor-acceptor fluorophores. H-Probe could self-assemble into stable nanomicelles in aqueous phase. Under normoxia, the FAM fluorescence was originally quenched by Dabcyl *via* FRET process. However, the tumor hypoxia would terminate the FRET process with the fluorescence recovery of FAM for hypoxia imaging. Moreover, in the presence of oxygen and light irradiation, H-Probe could generate the singlet oxygen for robust photodynamic therapy in incubation time- and concentration-dependent manners. After intravenous injection, H-Probe-induced photodynamic therapy could efficiently restrain the tumor growth with a minimized side effect. This FRET-based simultaneous hypoxia imaging and photodynamic therapy strategy would inspire the development of constructing theranostic nanoplatform for tumor precision therapy.

## Experiment

### Material

Rink amine resin (0.38 mmol/g), N-fluorenyl-9-methoxycarbonyl (Fmoc)-protected amino acid of Fmoc-Lys(Dabcyl)-OH and Fmoc-Lys(Mtt)-OH, 1-hydroxybenzotriazole (HOBt), o-benzotriazole-N,N,N′,N′-tetramethyluroniumhexaflorophosphate (HBTU) were obtained from GL Biochem (Shanghai, China) Ltd. Mito-Tracker Green, 3,3′-dioctadecyloxacarbocyanine perchlorate (DiO), singlet oxygen sensor green (SOSG), annexin V-FITC, Hoechst 33342 and 2′,7′-dichloroflorescein diacetate (DCFH-DA) were provided by Beyotime (China). Fmoc-NH-PEG_8_-CH_2_CH_2_COOH was provided by Biomatrik Inc. Hydrazine hydrate, trifloroacetic acid (TFA), diisopropylethylamine (DIEA), Protoporphyrin IX (PpIX), 5(6)-Carboxyfluorescein (FAM) and triisopropylsilane (TIS) were purchased from Aladdin Reagent Co. Ltd. (China). LysoTracker Green, Cell Counting Kit (CCK-8) and Calcein-AM/PI Double Stain Kit were purchased from Yeasen Biotech Co. Ltd. (China). Dulbecco's modified Eagle's medium (DMEM), Dulbecco's phosphate buffered saline (PBS), fetal bovine serum (FBS), antibiotics, and penicillin-streptomycin were provided by Invitrogen Corp.

The molecular weight was measured by electrospray ionization-mass spectrometry (ESI-MS, Finnigan LCQ advantage). Particle size was analyzed by Nano-ZS ZEN 3600 (Malvern) and transmission electron microscopy (TEM, JEOL-1400 PLUS). The zeta potential was detected by NanoBrook 90Plus Zeta (Brookhaven). Inverted microscope was applied to picture the H&E staining of tissues. Fluorescence was recorded by LS55 luminescence spectrometer (Perkin-Elmer). UV-vis absorbance was recorded by UV-vis spectrophotometry Lambda 35 (Perkin-Elmer). The intracellular fluorescence was shown by confocal laser scanning microscope (CLSM, LSM 880, Carl Zeiss). The *in vitro* and *in vivo* photodynamic therapy (PDT) was conducted using 630 nm LED light (power intensity: 29.8 mW cm^−2^) and 630 nm He-Ne laser (power intensity: 320 mW cm^−2^), respectively. The cell viability was measured by microplate reader (Bio-Rad) and flow cytometry (Amnis, Merck millipore).

### Synthesis and Characterization of H-Probe

H-Probe was synthesized by using standard solid phase peptide synthesis (SPPS) method as pervious reports and it was stocked at −20°C in the shield of light for later use. The molecular weight of H-Probe was characterized by ESI-MS. The particle size of H-Probe in water, PBS or 10% FBS/PBS containing 0.1% DMSO was analyzed by Nano-ZS ZEN 3600. And the zeta potential was detected by NanoBrook 90Plus Zeta. The morphology of H-Probe was observed by TEM. Moreover, the UV-vis absorbance of H-Probe at the concentration of 25 μM was recorded by UV-vis spectrophotometry.

### Cell Culture

Murine mammary carcinoma (4T1) cells were incubated in DMEM medium containing 10% FBS and 1% antibiotics in an atmosphere of 5% CO2 at 37°C.

### Fluorescence Recovery Measurement

Above all, in the presence or absence of sodium hydrosulfite, the FAM fluorescence recovery of H-Probe in PBS (200 mM, pH 7.4) containing 1% DMSO was monitored by using LS55 luminescence spectrometer. Moreover, the cellular fluorescence recovery of H-Probe was also detected by CLSM and flow cytometry. Briefly, 4T1 cells were seeded and cultured for 24 h. Then the cells were treatment with H-Probe (50 μM) for another 24 h in normoxia (21% O_2_) or hypoxia (1% O_2_). On the one hand, the cells were washed with PBS for CLSM observation. On the other hand, the cells were washed and collected for flow cytometry analysis.

### ROS Detection

In this work, the ROS generation was detected by using fluorescence spectrometer, CLSM and flow cytometry. For fluorescence detection, SOSG was used as the sensor of ROS. In brief, in the presence or absence of light irradiation, H-Probe (30 μM) was incubated with SOSG (5 μM) in PBS under normoxia (21% O_2_) or hypoxia (1% O_2_). PBS mixed with SOSG (5 μM) was used as the control. At the predetermined time, the fluorescence intensity of H-Probe was recorded. Additionally, after treatment with SOSG (5 μM), the fluorescence changes of H-Probe (30 μM) were also measured every minute under light irradiation or in the shield of light at the scheduled time.

For CLSM and flow cytometry analysis, DCFH-DA was used as the indicator of ROS. In brief, 4T1 cells were seeded and cultured for 24 h. Then the cells were treatment with H-Probe (30 μM) for another 4 h in normoxia (21% O_2_) or hypoxia (1% O_2_). After washed with PBS, the cells were incubated with DCFH-DA (10 μM) for 20 min. With or without light irradiation (5 min), the fluorescence in 4T1 cells was imaged by CLSM. For flow cytometry analysis, 4T1 cells were treated with H-Probe (5 μM) for 4 h in normoxia (21% O_2_) or hypoxia (1% O_2_). Then the cells were washed, collected and then incubated with DCFH-DA for 20 min. After that, the cells were exposed to light for 200 s or incubated in the dark. At last, the intracellular fluorescence was analyzed by flow cytometry.

### Cellular Uptake and Subcellular Localization

Firstly, 4T1 cells were seeded and cultured for 24 h. Then, the cells were incubated with H-Probe (50 μM) for 4 h. After washed with PBS, the cells were stained with Hoechst 33342, LysoTracker Green and MitoTracker Green for 20, 15, 30, and 20 min, respectively. Subsequently, the cells washed and observed by CLSM.

### Cytotoxicity of H-Probe

The cytotoxicity of H-Probe was evaluated by CCK-8 assay, live/dead cell staining assay, cell apoptosis assay and trypan blue staining assay. For CCK-8 assay, 4T1 cells were seeded in 96 -well plates and cultured for 24 h. Then the cells were incubated with gradient concentrations of H-Probe for 12 h under normoxia (21% O_2_) or hypoxia (1% O_2_). Then the cells were exposed to LED light for 200 s (630 nm, power intensity: 29.8 mW/cm^2^) or incubated in the dark. After another 12 h, 10 μL of CCK-8 was added into every well. Four hours later, the absorbance at 450 nm was detected by the microplate reader. The relative cell viability was calculated as follows: cell viability (%) = OD (sample) × 100/OD (control), where OD (control) and OD (sample), respectively, represented the absorbance of samples in the absence and presence of H-Probe.

For live/dead cell staining assay, 4T1 cells were seeded and cultured for 24 h. Subsequently, the cells were treated with H-Probe (50 μM) for 4 h under normoxia (21% O_2_) or hypoxia (1% O_2_). After that, the cells were exposed to light for 1 min or incubated in the dark. Lastly, the cells were stained with Calcein-AM/ PI for 20 min and then observed by CLSM.

For cell apoptosis assay, 4T1 cells were seeded and cultured for 24 h. Then the cells were treated with H-Probe (10 μM) for 4 h under normoxia (21% O_2_) or hypoxia (1% O_2_). Subsequently, the cells were irradiated for 1 min or incubated in the dark. After that, the cells was stained with Annexin V-FITC/PI for 15 min. Then the cell apoptosis was analyzed by flow cytometry.

### *In vivo* Tumor Therapy

All of the *in vivo* experiments were performed according to the guidelines of the Institutional Animal Care and Use Committee (IACUC) of the Animal Experiment Center of Guangzhou Medical University (Guangzhou, China) as well as the Regulations for the Administration of Affairs Concerning Experimental Animals. *In vivo* tumor model was established by subcutaneously injecting 4T1 cells into the hind leg region of female BALB/c mice. 4T1 tumor-bearing mice were divided into three groups randomly (5 mice in each group), including PBS group, H-Probe group and H-Probe with light irradiation group. 4T1 tumor-bearing mice were intravenously injected with 200 μL of H-Probe for PDT at an equivalent PpIX concentration of 3 mg/kg per mouse. After administration for 12 h, the mice in light group were exposed to 630 nm He-Ne laser (power intensity: 320 mW cm^−2^) for 5 min. The tumor volume and body weight of the mice were monitored every other day during the treatments. Tumor volume (V) was calculated as follows: V = (tumor width)^2^ × (tumor length)/2. After 13 days, the mice were sacrificed and the tumors were obtained for weighing and photographing. Moreover, the tumors and the main organs (heart, liver, spleen, lung, kidney) were also analyzed through hematoxylin/eosin (H&E) staining. Besides, the blood biochemistry and blood routine of the mice were also analyzed after treatment with PBS or H-Probe for 13 days.

## Result and Discussion

### Preparation and Characterization of H-Probe

**Figure 1:**
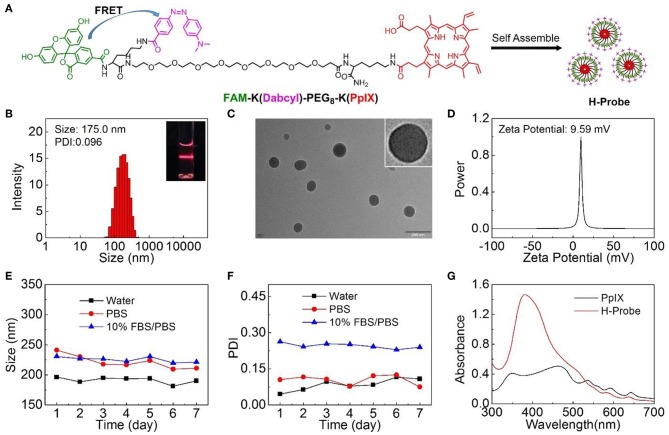
Preparation and characterizations of H-Probe. **(A)** The chemical structure of FAM-K(Dabcyl)-PEG_8_-K(PpIX) and the preparation of H-Probe. **(B)** The size distribution of H-Probe. Insert: Dindall effect of H-Probe solution. **(C)** TEM images and magnified TEM image of H-Probe. Scale bar: 200 nm. **(D)** Zeta potential of H-Probe. **(E)** The particle size and **(F)** PDI changes of H-Probe in 7 days. **(G)** The UV-vis absorbance of H-Probe and PpIX.

H-Probe was synthesized by solid phase synthesis method. The concrete laboratory procedure was shown in Supplementary Figure 1. The structure of H-Probe was verified by ESI-MS (Supplementary Figure 2). As illustrated in Figure 1A, the fluorescence of FAM was expected to be quenched by Dabcyl through the FRET process. The participation of PEG facilitated the self-assembly of H-Probe into nanomicelles. As suggested in Figure 1B, H-Probe exhibited a relatively narrow particle size distribution and good Tyndall effect, which indicated a high dispersion. Moreover, the morphology of H-Probe was also observed by TEM (Figure 1C), which further confirmed the effective self-assembly of H-Probe into spherical nanoparticles in aqueous phase. Additionally, the zeta potential of H-Probe was determined to be positive (Figure 1D), which was beneficial for its cellular uptake. To evaluate the stability, the changes of particle size (Figure 1E) and the PDI (Figure 1F) of H-Probe in water, PBS and PBS containing 10% FBS were monitored in 1 week. Particularly, the particle size and PDI of H-Probe in 10% FBS/PBS was found to be larger than that of in water, which might be ascribed to the inevitable adsorption of positive H-Probe to serum. Even so, they still remained relatively stable, which was important for its following hypoxia imaging. Moreover, the UV-vis absorbance of H-Probe and PpIX was also characterized as shown in Figure 1G. Compared with PpIX, the sharp Soret band around 400 nm indicated the weakened π-π stacking induced aggregation, demonstrated that the modification of PEG could improve the physicochemical property of PpIX. Due to the improved hydrophobic and aggregation behavior of PpIX, H-Probe would possess an enhanced stability the solubility, which would be favorable to generate ROS for PDT against hypoxic tumor.

### Hypoxia Imaging Ability of H-Probe

**Figure 2:**
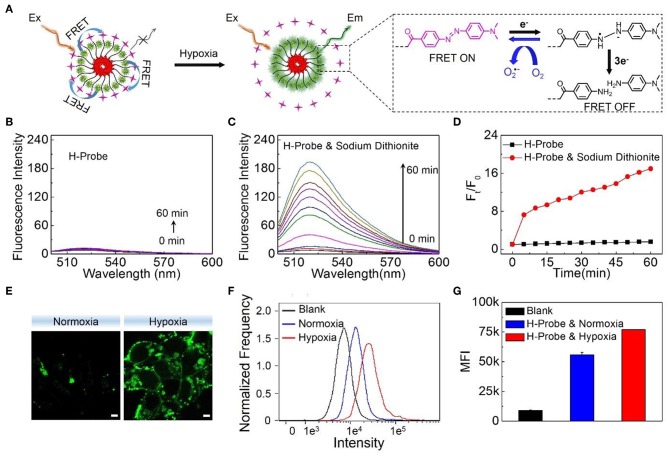
Hypoxia imaging ability of H-Probe. **(A)** Schematic illustration and the proposed mechanism of H-Probe for hypoxia imaging. Real time fluorescence spectra of H-Probe in the **(B)** absence or **(C)** presence of sodium dithionite in 60 min. **(D)** Fluorescence changes of H-Probe in the presence or absence of sodium dithionite. **(E)** CLSM images of 4T1 cells after treatment with H-Probe under normoxia or hypoxia conditions. Scale bar: 5 μm. **(F)** Flow cytometry and **(G)** the quantitative fluorescence analysis of 4T1 cells after treatment with H-Probe under normoxia or hypoxia conditions.

Under the normoxic condition, the FRET between FAM and Dabcyl could induce the fluorescence quenching. However, the azobenzene bond in H-Probe might be reduced under hypoxic condition, resulting in the interruption of the FRET process with the fluorescence recovery of FAM (Figure 2A). Significantly, H-Probe demonstrated a great potential for hypoxia imaging. To confirm it, sodium dithionite as a chemical mimic of azoreductase was used to break the azobenzene bond and terminate the FRET process. Above all, the fluorescence spectra of H-Probe was recorded in the absence of sodium dithionite. As shown in Figure 2B, the fluorescence intensity was found to be very low due to the FRET-induced fluorescence quenching. What's more, no obvious fluorescence changes were observed in 60 min, implying the good stability of H-Probe. However, with the addition of sodium dithionite, the fluorescence intensity was enhanced significantly in a time-dependent manner (Figure 2C), which was further confirmed by the relative fluorescence changes (Figure 2D). These investigations demonstrated that the reduction of the azobenzene bond would really interrupt the FRET and recover the fluorescence of FAM. To determine whether the hypoxia could also trigger the fluorescence recovery, 4T1 cells were treated with H-Probe under normoxia or hypoxia for CLSM observations. As displayed in Figure 2E, stronger green fluorescence was found in the cells under hypoxia rather than that of under normoxia, which verified the hypoxia-induced FAM fluorescence recovery. Similar result was also confirmed by the quantitative flow cytometry analysis (Figures 2F,G). Based on the above, it could be concluded that H-Probe could be applied for hypoxia imaging by reducing the azobenzene bond and interrupting the FRET to recover the FAM fluorescence.

### ROS Generation by H-Probe

**Figure 3:**
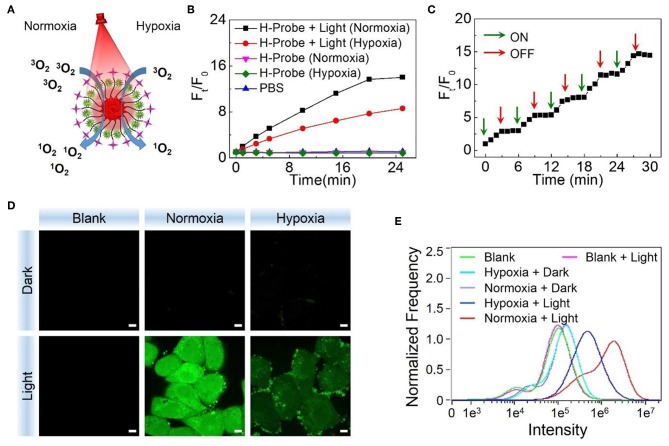
PDT potential of H-Probe. **(A)** Schematic illustration of H-Probe for PDT under normoxia and hypoxia conditions. **(B)** Time related SOSG fluorescence changes of H-Probe under normoxia or hypoxia conditions in the presence or absence of light irradiation. **(C)** Singlet oxygen production of H-Probe under ON-OFF light irradiation. **(D)** CLSM images and **(E)** Flow cytometry analysis of 4T1 cells after treatment with H-Probe under normoxia or hypoxia conditions in the presence or absence of light irradiation. Scale bar: 5 μm.

**Figure 4:**
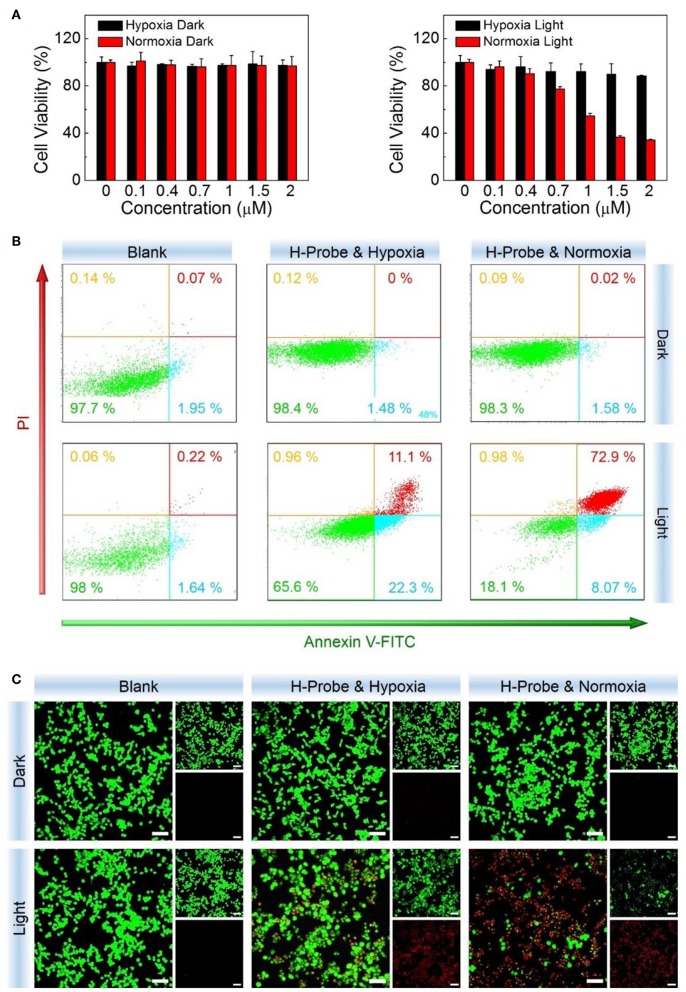
PDT studies of H-Probe *in vitro*. Anti-proliferation ability of H-Probe against 4T1 cells under normoxia or hypoxia conditions in the presence or absence of light irradiation measured by **(A)** CCK-8 kit, **(B)** flow cytometry, and **(C)** live/dead cells staining assay. Scale bar: 5 μm.

After verifying the hypoxia imaging ability, the ROS generation of H-Probe under normoxia or hypoxia was evaluated subsequently. As illustrated in Figure 3A, singlet oxygen (^1^O_2_) as a kind of toxic ROS, could be generated in the presence of light irradiation, photosensitizer and oxygen. When without light irradiation, negligible SOSG fluorescence changes were found after treatment with H-Probe in normoxia or hypoxia (Figure 3B), suggesting little production of ROS. Once exposed to the light, the fluorescence was obviously increased. H-Probe with light under normoxia exhibited stronger SOSG fluorescence than that of under hypoxia, implying the importance of oxygen for producing ROS. Besides, the SOSG fluorescence changes under ON-OFF irradiation indicated the controllability of H-Probe by light to generate ROS (Figure 3C), which highlighted the advantages of PDT for tumor therapy. Further, the cellular ROS generation was also assessed by CLSM and flow analysis. As shown in Figures 3D,E, 4T1 cells in the dark with various treatments exhibited ignorable fluorescence, reflecting the indispensability of light. Due to the limitation of oxygen, 4T1 cells treated with H-Probe and light under hypoxia still showed weak green fluorescence. Conversely, intense green fluorescence was observed in 4T1 cells under normoxia after treatment with H-Probe and light, indicating a great potential for PDT. Moreover, the flow analysis also reflected the similar phenomena, suggesting the light- and oxygen-related production of ROS by H-Probe.

### *In vitro* PDT Evaluation of H-Probe

Prior to evaluating the PDT effect of H-Probe *in vitro*, its cellular uptake behavior was investigated by CLSM. As displayed in Supplementary Figure 3, after treatment with 4T1 cells for 4 h, obvious red fluorescence of H-Probe was found in cells, indicating an efficient cellular uptake. Besides, there was little overlap between the red H-Probe fluorescence and the blue Hoechst 33342 fluorescence, illustrating that the internalized H-Probe mainly existed in cytoplasm. Furthermore, H-Probe was found to overlay well with LysoTracker Green while separate with MitoTracker Green, implying that H-Probe was internalized into cells by cellular endocytosis pathway.

Subsequently, the internalized H-Probe induced cytotoxicity was evaluated by CCK-8 assay. Nanoprobes for hypoxia imaging should have good biocompatibility with a low cytotoxicity. Based on this, the dark toxicity of H-Probe was firstly detected. As shown in Figure 4A, regardless of the oxygen content, H-Probe had very low cytotoxicity, which was favorable for hypoxia imaging. Moreover, owing to the restriction of oxygen, H-Probe in hypoxia also caused a weak PDT effect under light irradiation. However, H-Probe in normoxia exhibited a concentration-dependent photo toxicity, suggesting a robust PDT effect. In addition, the cytotoxicity of H-Probe was also assessed by cell apoptosis assay. As displayed in Figure 4B, when without light irradiation, very few cells was in apoptosis state. Even after adding the light, H-Probe under hypoxia only triggered about a third of 4T1 cells apoptosis, reflecting a limited PDT effect. However, after improving the oxygen content, more than 80% of the cells were apoptotic after treatment with H-Probe and light under normoxia. Similar results were also observed by the live/dead cell staining assay (Figure 4C). Intense red fluorescence and very weak green fluorescence were found in 4T1 cells treated with H-Probe and light under normoxia, demonstrating the best PDT effect against 4T1 cells.

### *In vivo* Anti-tumor Study of H-Probe

**Figure 5:**
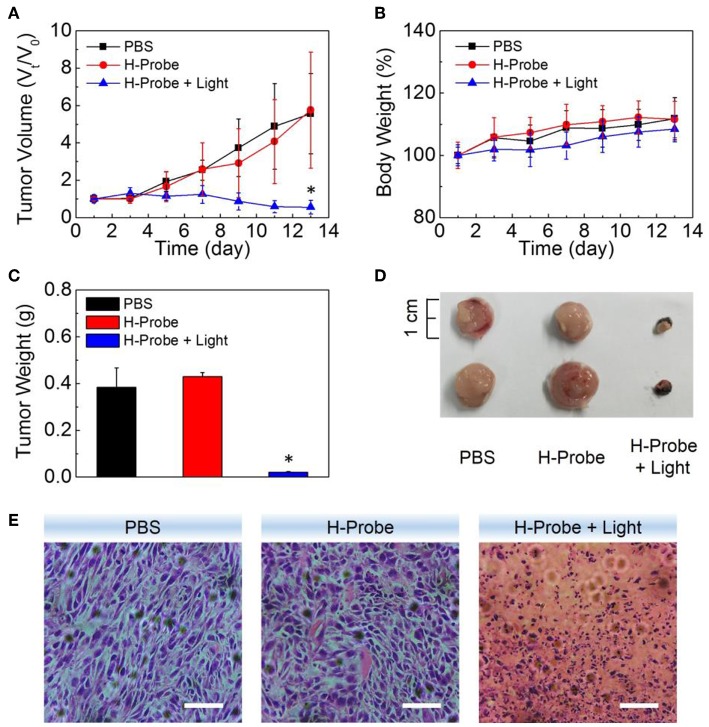
Anti-tumor studies of H-Probe *in vivo*. **(A)** The relative tumor volume and **(B)** the body weight changes of the mice after treatment with PBS, H-Probe, H-Probe and light in 13 days. V_0_ and M_0_ represented the tumor volume and body weight of the mice at the first day when without any treatments. **(C)** The average tumor weight, **(D)** the represented tumor tissues and **(E)** the H&E staining images of the sacrificed tumor tissues at the 13th day.

**Figure 6:**
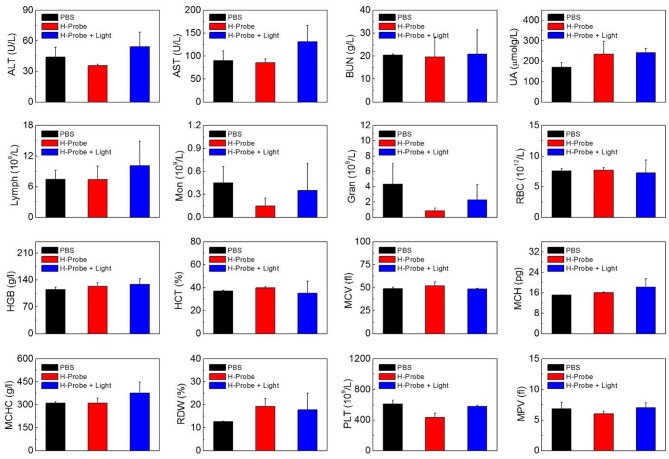
Biocompatibility of H-Probe *in vivo*. ALT, AST, BUN, UA analysis and the hematological parameters of the mice after treatment with PBS, H-Probe, H-Probe, and light at the 13th day.

To further investigate the anti-tumor effect *in vivo*, H-Probe was intravenously injected into the 4T1 tumor-bearing mice for PDT. As indicated in Figure 5A, just like PBS, H-Probe without light could scarcely inhibited the tumor growth. But H-Probe-induced PDT exhibited an excellent therapeutic effect on 4T1 tumor. Not only that, the relative body weight kept increasing slowly during the treatment (Figure 5B). These results highlighted the advantages of H-Probe for the effective PDT with a low system toxicity *in vivo*. After treatment with 13 days, the tumors were harvested for weighting and photographing. As shown in Figures 5C,D, compared with PBS and H-Probe without light, H-Probe with light significantly inhibited the tumor growth. Besides, the tumor tissues were also sliced for H&E staining analysis. As displayed in Figure 5E, tumor tissues of the mice treated with PBS or H-Probe without light were mainly filled with lots of tumor cells, which exhibited no obvious damage. However, there were many cells without nuclei or with destroyed nuclei in tumors treated with H-Probe and light, suggesting a robust PDT against tumors. Furthermore, the normal tissues of heart, liver, spleen, lung and kidney were also performed for H&E staining (Supplementary Figure 4). After treatment with H-Probe and light, the morphologies of cells in these tissues were basically normal, indicating no apparent toxicity of this PDT to main organs. However, it should be noted that an obvious tumor metastasis was found in liver of the mice treated with PBS or H-Probe without light due to the ineffective treatments. What's more, the blood biochemistry and blood routine of the mice were also carried out to evaluate the biocompatibility of H-Probe. As illustrated in Figure 6, although there were some differences in the detection results of the mice between various groups, they were still in the normal range, verifying that H-Probe possessed good biocompatibility and it caused no significant system toxicity. Based on the above, it could be concluded that H-Probe realized a robust PDT *in vivo* under light irradiation to inhibit the tumor growth and metastasis with a minimized side effect.

## Conclusion

In summary, we designed a smart theranostic nanoprobe for hypoxia imaging and photodynamic tumor therapy. Owing to the FRET process, the fluorescence of FAM could be effectively quenched by Dabcyl in the nanoprobe. However, an effective reduction of azobenzene bond in Dabcyl would terminate the FRET process with a significant fluorescence recovery of FAM, which was able to be used for hypoxia imaging *in vitro*. Furthermore, the photosensitizer of PpIX in nanoprobe could achieve an efficient PDT against hypoxic tumor with a low side effect. In a word, based on FRET, this nanoprobe realized not only the hypoxia imaging but also the photodynamic tumor therapy, which provided a new insight for developing theranostic nanoplatform in tumor precision therapy.

## Data Availability Statement

All datasets generated for this study are included in the article/Supplementary Material.

## Ethics Statement

The animal study was reviewed and approved by Institutional Animal Care and Use Committee (IACUC) Animal Experiment Center of Guangzhou Medical University (Guangzhou, China).

## Author Contributions

JF prepared materials and carried out *in vivo* experiments. GF helped to perform *in vitro* experiments. PY helped to characterize materials and incubate cells. FD and XZ helped to characterize materials. LL helped to culture cells. XY supervised the work. HC supervised the work and wrote the manuscript. SL provided an idea and designated the whole research.

### Conflict of Interest

The authors declare that the research was conducted in the absence of any commercial or financial relationships that could be construed as a potential conflict of interest.
