# Supplementary material for: A Theranostic Nanoprobe for Hypoxia Imaging and Photodynamic Tumor Therapy
Source: Front Chem. 2019 Dec 20;7:868. doi: 10.3389/fchem.2019.00868 (PMC6933523; doi:10.3389/fchem.2019.00868)
Supplement: Supplementary file 1 [file Table_1.DOCX]

Supplementary Material

**A Theranostic Nanoprobe for Hypoxia Imaging and Photodynamic Tumor Therapy**

**Jing Hao Fan^1^, Gui Ling Fan^1^, Ping Yuan^1^, Fu An Deng^1^, Ling Shan Liu^2^, Xiang Zhou^2^, Xi Yong Yu^1*^, Hong Cheng^2*^ and Shi Ying Li^1*^**

^1^Key Laboratory of Molecular Target & Clinical Pharmacology and the State Key Laboratory of Respiratory Disease, School of Pharmaceutical Sciences & The Fifth Affiliated Hospital, Guangzhou Medical University, Guangzhou 511436, PR China.

^2^Guangdong Provincial Key Laboratory of Construction and Detection in Tissue Engineering, Biomaterials Research Center, School of Biomedical Engineering, Southern Medical University, Guangzhou 510515, PR China.

## Supplementary Figures


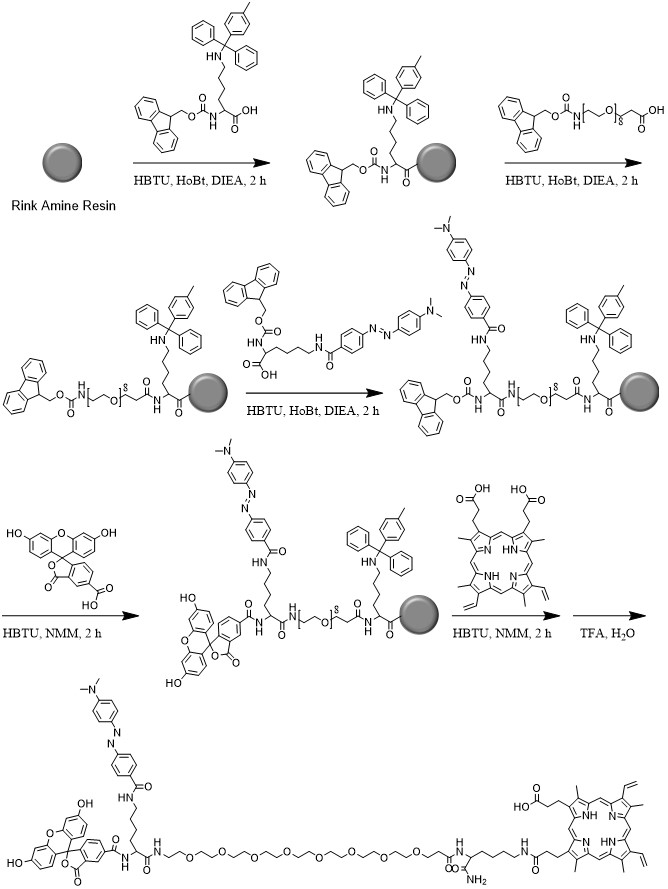


**Supplementary Figure 1.** Detailed synthetic procedure of FAM-K(Dabcyl)-PEG_8_-K(PpIX).

**Supplementary Figure 2.** ESI-MS of H-Probe.


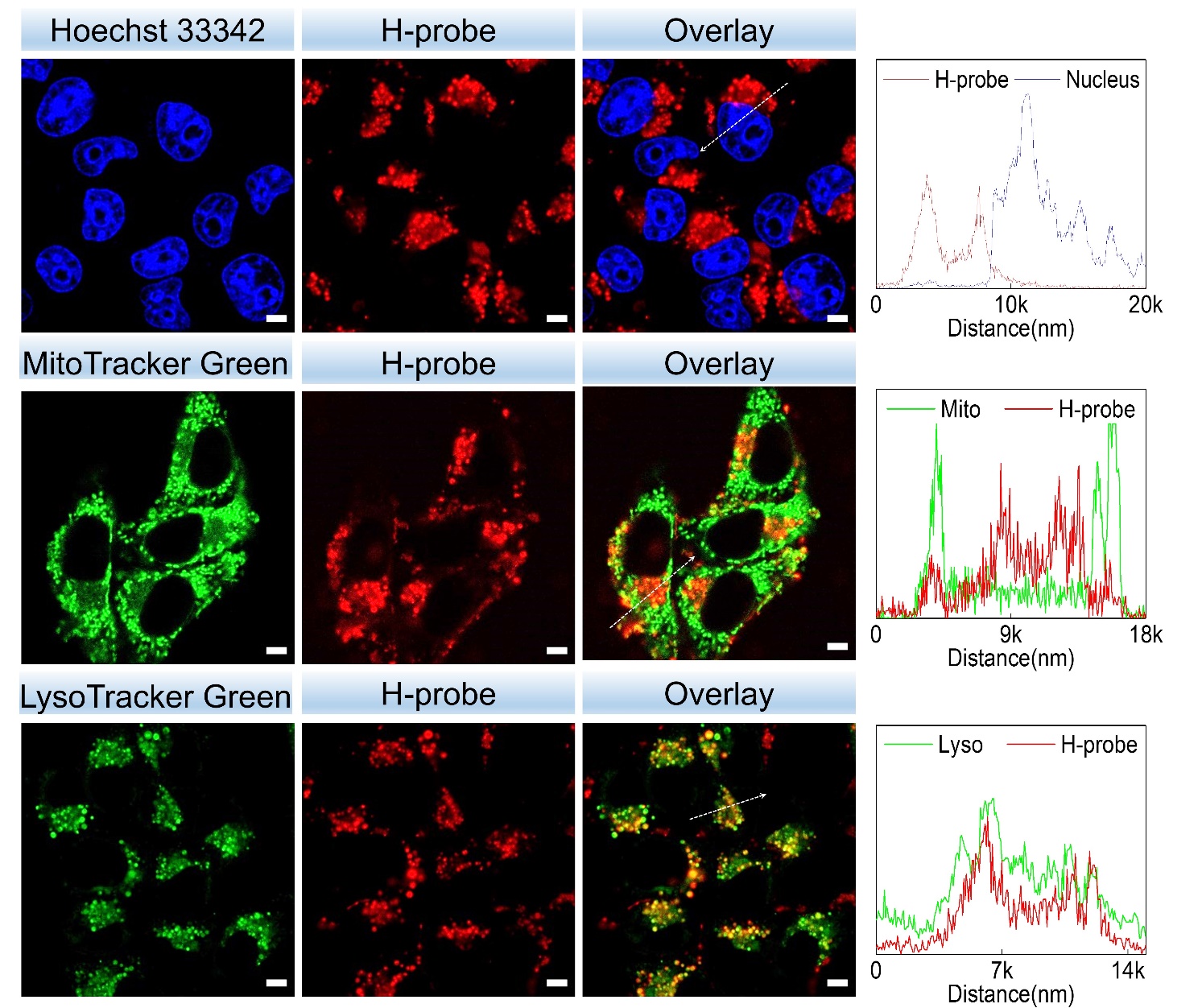


**Supplementary Figure 3.** CLSM images and fluorescence profile analysis of 4T1 cells after treatment with H-Probe and stained by Hoechst 33342, MitoTracker Green or LysoTracker Green. Scale bar: 5 µm.


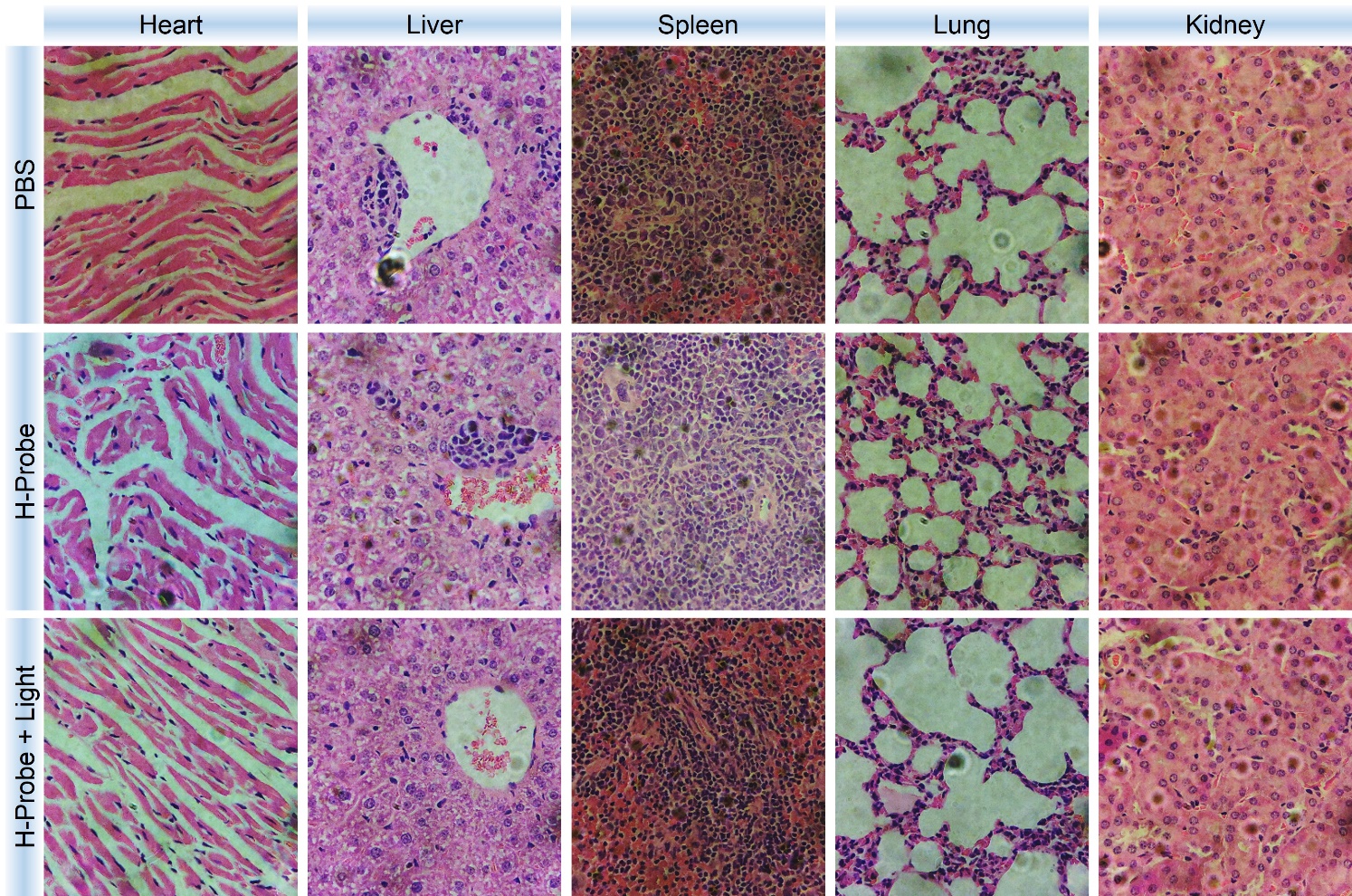


**Supplementary Figure 4.** H&E staining analysis of the sacrificed heart, liver, spleen, lung and kidney after various treatments at the 13^th^ day.
